# The genome sequence of the meadow field syrph, *Eupeodes latifasciatus* (Macquart, 1829)

**DOI:** 10.12688/wellcomeopenres.18113.1

**Published:** 2022-10-12

**Authors:** Steven Falk, University of Oxford and Wytham Woods Genome Acquisition Lab, Darwin Tree of Life Barcoding collective, Wellcome Sanger Institute Tree of Life programme, Wellcome Sanger Institute Scientific Operations: DNA Pipelines collective, Tree of Life Core Informatics collective, Physilia Chua, Darwin Tree of Life Consortium

**Affiliations:** 1 Independent researcher, Kenilworth, Warwickshire, UK; 2 Wellcome Trust Sanger Institute, Hinxton, UK

**Keywords:** Eupeodes latifasciatus, meadow field syrph, genome sequence, chromosomal, Lepidoptera

## Abstract

We present a genome assembly from an individual female *Eupeodes latifasciatus* (meadow field syrph; Arthropoda; Insecta; Diptera; Syrphidae). The genome sequence is 846 megabases in span. The majority of the assembly (96.8%) is scaffolded into 4 chromosomal pseudomolecules with the X sex chromosome assembled. The complete mitochondrial genome was also assembled and is 18.5 kilobases in length. Gene annotation of this assembly on Ensembl has identified 12,848 protein coding genes.

## Species taxonomy

Eukaryota; Metazoa; Ecdysozoa; Arthropoda; Hexapoda; Insecta; Pterygota; Neoptera; Endopterygota; Diptera; Brachycera; Muscomorpha; Syrphoidea; Syrphidae; Syrphinae; Syrphini; Eupeodes; *Eupeodes*; *Eupeodes latifasciatus* (Macquart, 1829) (NCBI:txid1124558).

## Background

The meadow field syrph, *Eupeodes latifasciatus*, is a type of hoverfly from the Syrphid family. Its wingspan is between 6.5 to 8.5 mm. It is similar to another hoverfly species, *Eupeodes corollae*, but it can be distinguished from the yellow markings on its body which are fused into bands on segments three and four ( van Veen, 2004).

*E. latifasciatus* can be found across the Palaearctic from the south of Fennoscandia to the Mediterranean basin ( Peck, 1988). It is widespread in the UK but occurs more frequently in the south, preferring lush vegetation and damp meadows to gardens ( Eupeodes Latifasciatus (Macquart, 1829) n.d.). Some of the common flowers that *E. latifasciatus* visits are white umbellifers, *Euphorbia*, and *Ranunculus* ( de Buck, 1990). While adults feed only on nectar, *E. latifasciatus* larvae feed on small insects from the insect order Hemiptera such as aphids and scale insects ( Stubbs & Falk, 1983). The flight period is usually from May to September but occurs from April to October in southern Europe. This high-quality *E. latifasciatus* genome was assembled as part of the Darwin Tree of Life project which aims to genetically describe all species found in the UK.

## Genome sequence report

**Figure 1.**
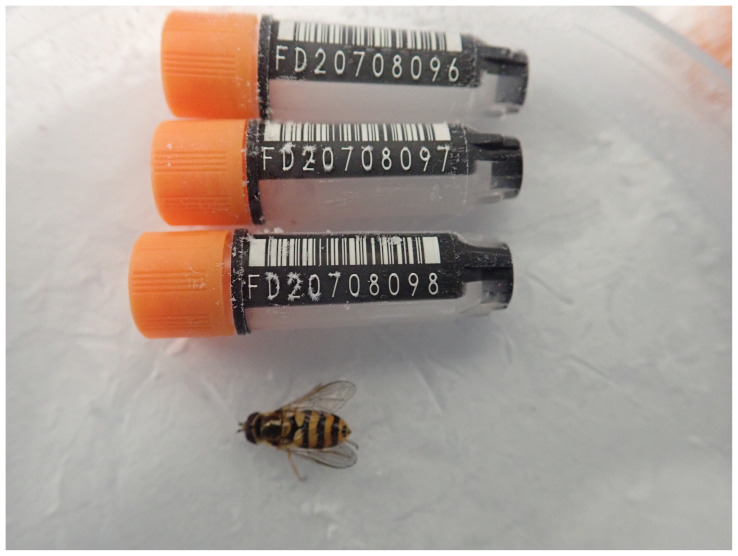
Image of the *Eupeodes latifasciatus* specimen taken prior to preservation and processing.

The genome was sequenced from a single female *E. latifasciatus* collected from Wytham Woods, Berkshire, UK ( Figure 1). A total of 27-fold coverage in Pacific Biosciences single-molecule HiFi long reads and 47-fold coverage in 10X Genomics read clouds were generated. Primary assembly contigs were scaffolded with chromosome conformation Hi-C data. Manual assembly curation corrected 492 missing/misjoins and removed 17 haplotypic duplications, reducing the assembly size by 1.20% and the scaffold number by 38.33%, and increasing the scaffold N50 by 437.36%.

**Figure 2.**
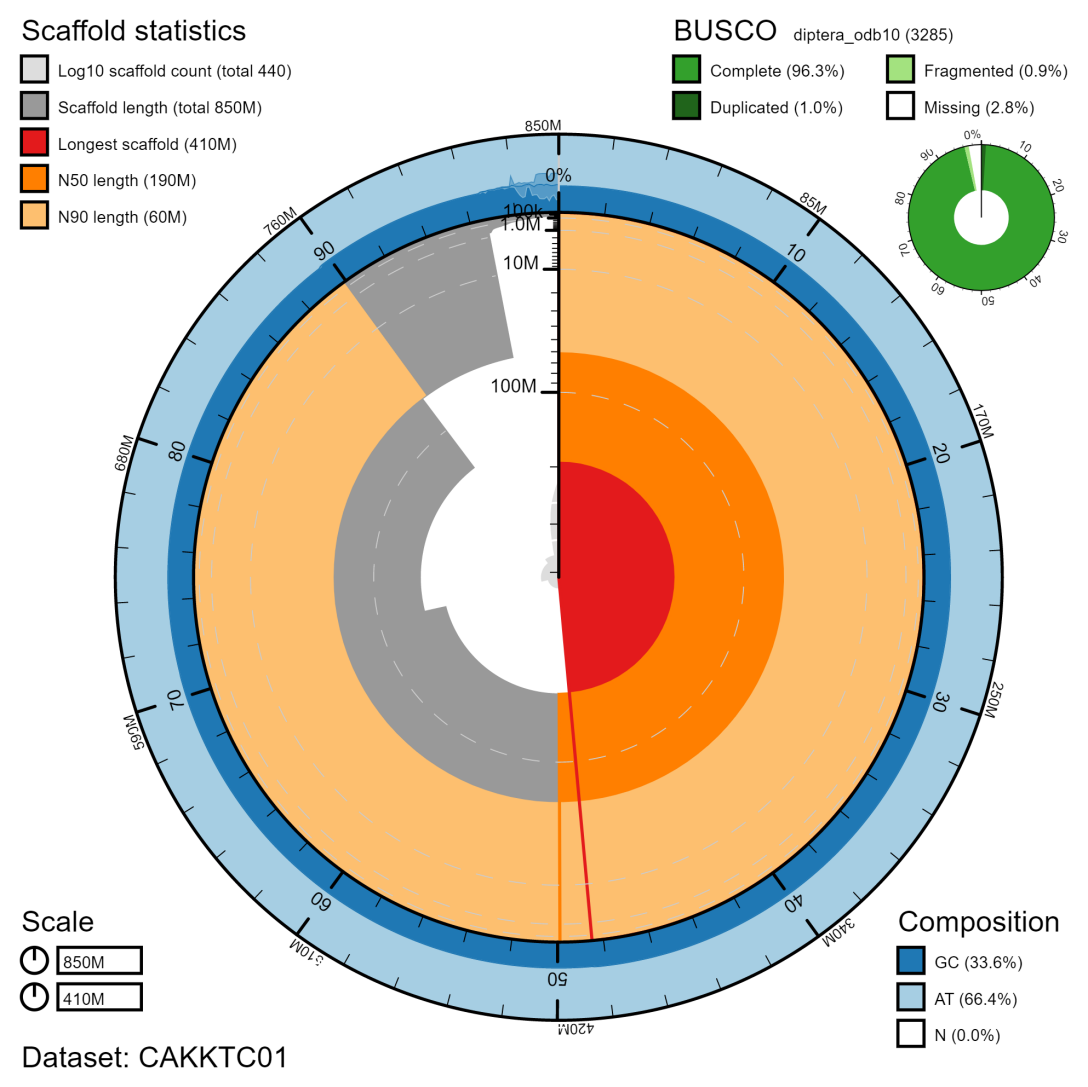
Genome assembly of *Eupeodes latifasciatus*, idEupLati1.1: metrics. The BlobToolKit Snailplot shows N50 metrics and BUSCO gene completeness. The main plot is divided into 1,000 size-ordered bins around the circumference with each bin representing 0.1% of the 846,356,614 bp assembly. The distribution of chromosome lengths is shown in dark grey with the plot radius scaled to the longest chromosome present in the assembly (410,988,561 bp, shown in red). Orange and pale-orange arcs show the N50 and N90 chromosome lengths (189,435,894 and 60,440,373 bp), respectively. The pale grey spiral shows the cumulative chromosome count on a log scale with white scale lines showing successive orders of magnitude. The blue and pale-blue area around the outside of the plot shows the distribution of GC, AT and N percentages in the same bins as the inner plot. A summary of complete, fragmented, duplicated and missing BUSCO genes in the diptera_odb10 set is shown in the top right. An interactive version of this figure is available at https://blobtoolkit.genomehubs.org/view/idEupLati1.1/dataset/CAKKTC01/snail.

The final assembly has a total length of 846 Mb in 436 sequence scaffolds with a scaffold N50 of 189.4 Mb ( Table 1). The majority, 96.8%, of the assembly sequence was assigned to 4 chromosomal-level scaffolds, representing 3 autosomes (numbered by sequence length) and the X sex chromosome ( Figure 2– Figure 5; Table 2). Two regions of this assembly are particularly fragmented: the centromeric and pericentromeric region of chromosome 1 and all of chromosome X.

**Table 1.** Genome data for *Eupeodes latifasciatus*, idEupLati1.1.

*Project accession data*	
Assembly identifier	idEupLati1.1
Species	*Eupeodes latifasciatus*
Specimen	idEupLati1 (genome assembly, Hi-C, RNA-Seq)
NCBI taxonomy ID	1124558
BioProject	PRJEB47320
BioSample ID	SAMEA7746776
Isolate information	Female. Thorax (genome assembly); head (Hi-C); abdomen (RNA-Seq)
*Raw data accessions*	
PacificBiosciences SEQUEL II	ERR6808042; ERR6939266
10X Genomics Illumina	ERR6688742-ERR6688745
Hi-C Illumina	ERR6688741
PolyA RNA-Seq Illumina	ERR9435022
*Genome assembly*	
Assembly accession	GCA_920104205.1
*Accession of alternate* * haplotype*	GCA_920104105.1
Span (Mb)	846
Number of contigs	1233
Contig N50 length (Mb)	2.7
Number of scaffolds	436
Scaffold N50 length (Mb)	189.4
Longest scaffold (Mb)	410.99
BUSCO * genome score	C:96.3%[S:95.3%,D:1.0%],F:0.9%, M:2.8%,n:3,285

*BUSCO scores based on the diptera_odb10 BUSCO set using v5.3.2. C= complete [S= single copy, D=duplicated], F=fragmented, M=missing, n=number of orthologues in comparison. A full set of BUSCO scores is available at https://blobtoolkit.genomehubs.org/view/idEupLati1.1/dataset/CAKKTC01/busco.

**Figure 3.**
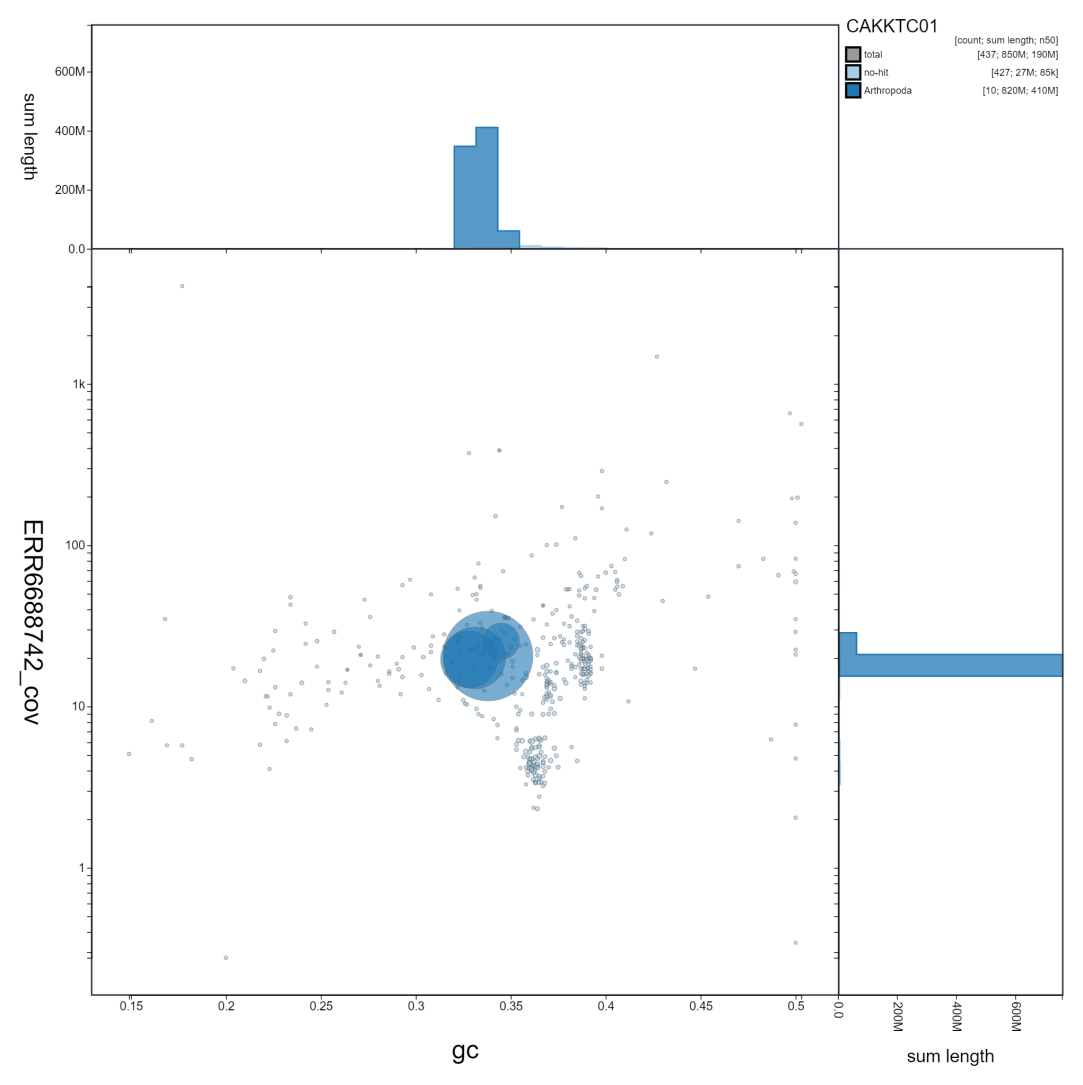
Genome assembly of *Eupeodes latifasciatus*, idEupLati1.1: GC coverage. BlobToolKit GC-coverage plot. Scaffolds are coloured by phylum. Circles are sized in proportion to scaffold length. Histograms show the distribution of scaffold length sum along each axis. An interactive version of this figure is available at https://blobtoolkit.genomehubs.org/view/idEupLati1.1/dataset/CAKKTC01/blob.

**Figure 4.**
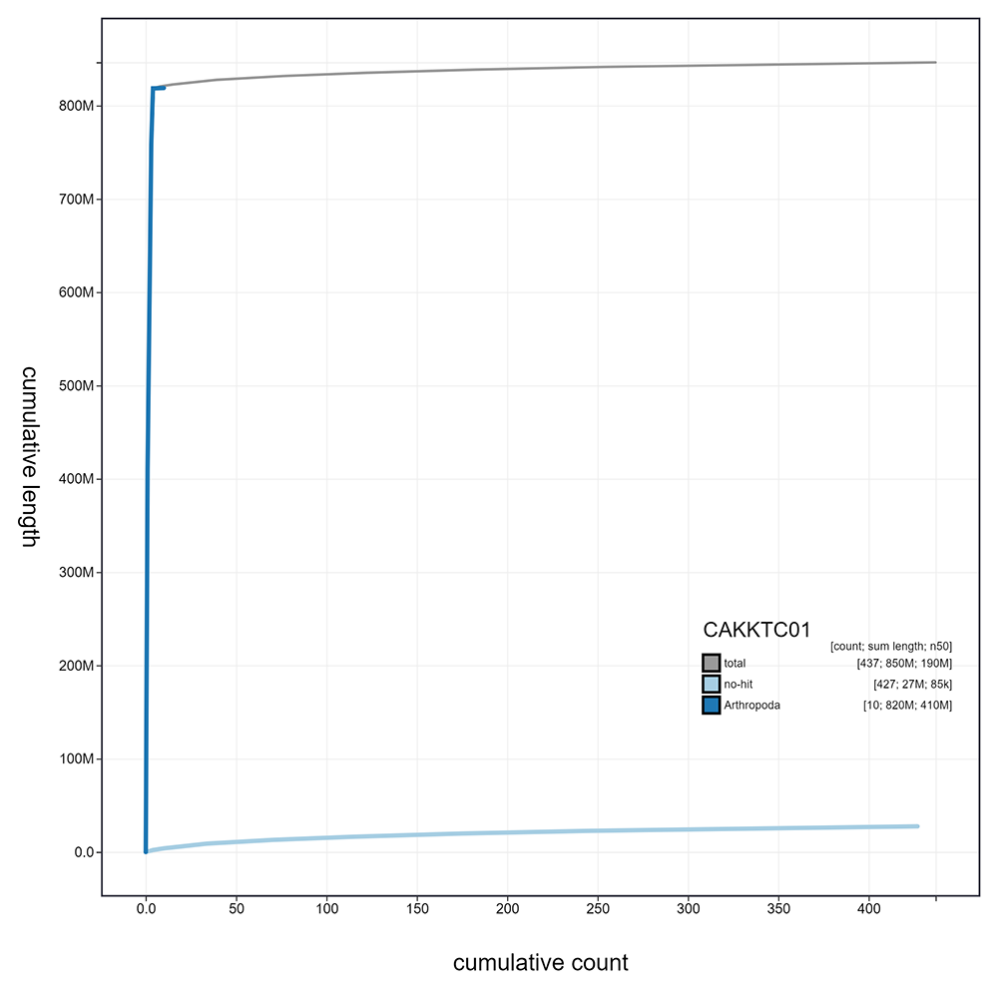
Genome assembly of *Eupeodes latifasciatus*, idEupLati1.1: cumulative sequence. BlobToolKit cumulative sequence plot. The grey line shows cumulative length for all scaffolds. Coloured lines show cumulative lengths of scaffolds assigned to each phylum using the buscogenes taxrule. An interactive version of this figure is available at https://blobtoolkit.genomehubs.org/view/idEupLati1.1/dataset/CAKKTC01/cumulative.

**Figure 5.**
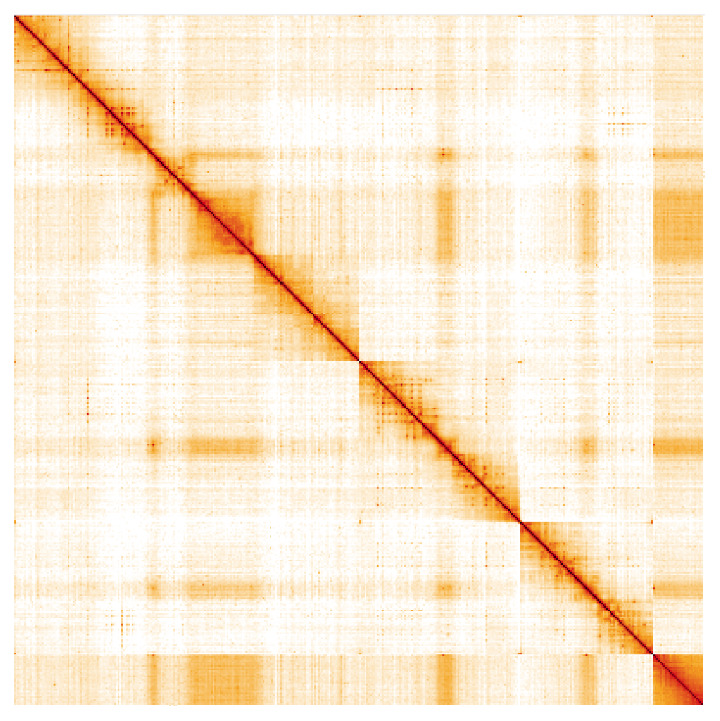
Genome assembly of *Eupeodes latifasciatus*, idEupLati1.1: Hi-C contact map. Hi-C contact map of the idEupLati1.1 assembly, visualised in HiGlass. Chromosomes are arranged in size order from left to right and top to bottom. The interactive Hi-C map can be viewed at https://genome-note-higlass.tol.sanger.ac.uk/l/?d=ZqciFudjRdakQ7z0HdB-xg.

**Table 2.** Chromosomal pseudomolecules in the genome assembly of *Eupeodes latifasciatus*, idEupLati1.1.

INSDC accession	Chromosome	Size (Mb)	GC%
OV049924.1	1	410.99	33.8
OV049925.1	2	157.65	32.8
OV049926.1	3	189.44	33.1
OV049927.1	X	60.44	34.5
OV049928.1	MT	0.02	17.7
-	Unplaced	27.83	36.1

The assembly has a BUSCO v5.3.2 ( Manni *et al*., 2021) completeness of 96.3% (single 95.3%, duplicated 1.0%) using the diptera_odb10 reference set (n=3,285). While not fully phased, the assembly deposited is of one haplotype. Contigs corresponding to the second haplotype have also been deposited.

## Genome annotation report

The idEupLati1.1 genome has been annotated using the Ensembl rapid annotation pipeline ( Table 1; https://rapid.ensembl.org/Eupeodes_latifasciatus_GCA_920104205.1/). The resulting annotation includes 21,916 transcribed mRNAs from 12,848 protein-coding and 2,996 non-coding genes.

## Methods

### Sample acquisition and nucleic acid extraction

A single female *E. latifasciatus* specimen (idEupLati1) was collected using a net from Wytham Woods, Berkshire, UK (latitude 51.769, longitude -1.339) by Steven Falk (University of Oxford). The specimen was identified by Steven Falk and snap-frozen on dry ice.

DNA was extracted at the Tree of Life Laboratory, Wellcome Sanger Institute. The idEupLati1 sample was weighed and dissected on dry ice with tissue set aside for Hi-C sequencing. Thorax tissue was disrupted using a Nippi Powermasher fitted with a BioMasher pestle. Fragment size analysis of 0.01-0.5 ng of DNA was then performed using an Agilent FemtoPulse. High molecular weight (HMW) DNA was extracted using the Qiagen MagAttract HMW DNA extraction kit. Low molecular weight DNA was removed from a 200-ng aliquot of extracted DNA using 0.8X AMpure XP purification kit prior to 10X Chromium sequencing; a minimum of 50 ng DNA was submitted for 10X sequencing. HMW DNA was sheared into an average fragment size between 12-20 kb in a Megaruptor 3 system with speed setting 30. Sheared DNA was purified by solid-phase reversible immobilisation using AMPure PB beads with a 1.8X ratio of beads to sample to remove the shorter fragments and concentrate the DNA sample. The concentration of the sheared and purified DNA was assessed using a Nanodrop spectrophotometer and Qubit Fluorometer and Qubit dsDNA High Sensitivity Assay kit. Fragment size distribution was evaluated by running the sample on the FemtoPulse system.

RNA was extracted from abdomen tissue of idEupLati1 in the Tree of Life Laboratory at the WSI using TRIzol, according to the manufacturer’s instructions. RNA was then eluted in 50 μl RNAse-free water and its concentration was assessed using a Nanodrop spectrophotometer and Qubit Fluorometer using the Qubit RNA Broad-Range (BR) Assay kit. Analysis of the integrity of the RNA was done using Agilent RNA 6000 Pico Kit and Eukaryotic Total RNA assay.

### Sequencing

Pacific Biosciences HiFi circular consensus and 10X Genomics Chromium read cloud sequencing libraries were constructed according to the manufacturers’ instructions. Sequencing was performed by the Scientific Operations core at the Wellcome Sanger Institute on Pacific Biosciences SEQUEL II (HiFi), Illumina NovaSeq 6000 (10X) and Illumina HiSeq 4000 (RNA-Seq) instruments. Hi-C data were generated in the Tree of Life laboratory from head tissue of idEupLati1 using the Arima v2 kit and sequenced on a NovaSeq 6000 instrument.

### Genome assembly

Assembly was carried out with Hifiasm ( Cheng *et al*., 2021); haplotypic duplication was identified and removed with purge_dups ( Guan *et al*., 2020). One round of polishing was performed by aligning 10X Genomics read data to the assembly with longranger align, calling variants with freebayes ( Garrison & Marth, 2012). The assembly was then scaffolded with Hi-C data ( Rao *et al*., 2014) using SALSA2 ( Ghurye *et al*., 2019). The assembly was checked for contamination as described previously ( Howe *et al*., 2021). Manual curation was performed using HiGlass ( Kerpedjiev *et al*., 2018) and Pretext. The mitochondrial genome was assembled using MitoHiFi ( Uliano-Silva *et al*., 2021), which performs annotation using MitoFinder ( Allio *et al*., 2020). The genome was analysed and BUSCO scores generated within the BlobToolKit environment ( Challis *et al*., 2020). Table 3 contains a list of all software tool versions used, where appropriate.

**Table 3.** Software tools used.

Software tool	Version	Source
Hifiasm	0.15.3	Cheng *et al*., 2021
purge_dups	1.2.3	Guan *et al*., 2020
SALSA2	2.2	Ghurye *et al*., 2019
longranger align	2.2.2	https://support.10xgenomics.com/ genome-exome/software/pipelines/ latest/advanced/other-pipelines
freebayes	1.3.1-17- gaa2ace8	Garrison & Marth, 2012
MitoHiFi	2.0	Uliano-Silva *et al*., 2021
HiGlass	1.11.6	Kerpedjiev *et al*., 2018
PretextView	0.2.x	https://github.com/wtsi-hpag/ PretextView
BlobToolKit	3.2.6	Challis *et al*., 2020

### Genome annotation

The Ensembl gene annotation system ( Aken *et al*., 2016) was used to generate annotation for the *Eupeodes latifasciatus* assembly (GCA_920104205.1). Annotation was created primarily through alignment of transcriptomic data to the genome, with gap filling via protein-to-genome alignments of a select set of proteins from UniProt ( UniProt Consortium, 2019).

### Ethics/compliance issues

The materials that have contributed to this genome note have been supplied by a Darwin Tree of Life Partner. The submission of materials by a Darwin Tree of Life Partner is subject to the Darwin Tree of Life Project Sampling Code of Practice. By agreeing with and signing up to the Sampling Code of Practice, the Darwin Tree of Life Partner agrees they will meet the legal and ethical requirements and standards set out within this document in respect of all samples acquired for, and supplied to, the Darwin Tree of Life Project. Each transfer of samples is further undertaken according to a Research Collaboration Agreement or Material Transfer Agreement entered into by the Darwin Tree of Life Partner, Genome Research Limited (operating as the Wellcome Sanger Institute), and in some circumstances other Darwin Tree of Life collaborators.

## Data Availability

European Nucleotide Archive: Eupeodes latifasciatus (meadow field syrph). Accession number PRJEB47320; https://identifiers.org/ena.embl/PRJEB47320. The genome sequence is released openly for reuse. The *E. latifasciatus* genome sequencing initiative is part of the Darwin Tree of Life (DToL) project. All raw sequence data and the assembly have been deposited in INSDC databases. Raw data and assembly accession identifiers are reported in Table 1.
