# Supplementary material for: The genome sequence of the meadow field syrph, Eupeodes latifasciatus (Macquart, 1829)
Source: Wellcome Open Res. 2022 Oct 12;7:253. [Version 1] doi: 10.12688/wellcomeopenres.18113.1 (PMC10015120; doi:10.12688/wellcomeopenres.18113.1)
Supplement: Peer review history

# Peer review: Reviewer response for version 1

**DOI:** 10.21956/wellcomeopenres.20084.r55470

**Published:** 2023-03-17

**Authors:** Darren J Obbard

**Affiliations:** 1 University of Edinburgh,, Edinburgh, UK

This data note reports the sequencing and assembly of the genome of *Eupeodes latifasciatus* as part of the “Darwin Tree of Life” programme. In common with other data notes from this research effort, the reporting is standardised and quite brief. As such, I have very few comments to make.

 The approach is state-of-the-art, the raw data appear to be of a suitably high quality, and the assembly methods are appropriate. The public availability of raw data and genome assembly are appropriate. The resulting genome is likely to be of very high quality, and I have no doubt that it will be of great value to any researchers working on this group of flies, or on the comparative or evolutionary genomics of insects more generally. The writing is clear, concise, and easy to read.

 Two issue already raised:

- I agree with the previous reviewer: “change 'type of hoverfly from the Syrphid family'. to 'species of hoverfly in the fly family Syrphidae.'”
- I agree with the previous reviewer: “added complementary information … In the section of Background:”. I would hope for another 2-3 more sentences on what basic biology is known (if any!), ideally citing 2-3 references that provide a links with the wider scientific literature.

Small textual suggestions:

- “assembly on Ensembl”: perhaps “assembly by Ensembl”, as I think Ensembl is a project, not a platform?
- “between 6.5 to 8.5 mm”: should probably either be “from X to Y” or “between X and Y”
- “The flight period is usually from May to September but occurs from April to October in southern Europe.” Could perhaps be “The flight period is usually from May to September in the UK and norther Europe, but is from April to October in southern Europe.”

Are sufficient details of methods and materials provided to allow replication by others?

Yes

Is the rationale for creating the dataset(s) clearly described?

Yes

Are the datasets clearly presented in a useable and accessible format?

Yes

Are the protocols appropriate and is the work technically sound?

Yes

Reviewer Expertise:

Population genetics, genomics, and phylogenetics of invertebrates and their pathogens

I confirm that I have read this submission and believe that I have an appropriate level of expertise to confirm that it is of an acceptable scientific standard.

# Peer review: Reviewer response for version 1

**DOI:** 10.21956/wellcomeopenres.20084.r55071

**Published:** 2023-03-14

**Authors:** Yokomi N Lozano-Sardaneta

**Affiliations:** 1 Universidad Nacional Autónoma de México,, Ciudad de México, Mexico

The authors present the genome assembly of the UK meadow *Eupeodes latifasciatus*. The analysis is clear and concise, with adequate description of: genome assembly, sequences and genome annotation.

 I would recommend added complementary information as follows:

 In the section of Background:

- I think it would be important to mention the biological relevance of this species, for example if it is pollinator, a pest, or serve as biological control ( *e.g*. aphids), etc.

In the section of Methods:

- I have a question, why was only one female analyzed and the male not included? Was it the only specimen you found? I consider it important to briefly explain why only one female specimen.

- Add the taxonomic keys used for Steven Falk for perform the taxonomic identification.

Are sufficient details of methods and materials provided to allow replication by others?

Yes

Is the rationale for creating the dataset(s) clearly described?

Yes

Are the datasets clearly presented in a useable and accessible format?

Yes

Are the protocols appropriate and is the work technically sound?

Yes

Reviewer Expertise:

insect taxonomy and phylogenetics

I confirm that I have read this submission and believe that I have an appropriate level of expertise to confirm that it is of an acceptable scientific standard.

# Peer review: Reviewer response for version 1

**DOI:** 10.21956/wellcomeopenres.20084.r54238

**Published:** 2023-02-21

**Authors:** Brian M. Wiegmann

**Affiliations:** 1 North Carolina State University,, Raleigh, USA

This is a fine description of a genome sequence, assembly and gene content assessment for the hoverfly species *Eupeodes latifasciatus*. The analyses are appropriate and clearly presented with good visualization of scaffold coverage, mapping and completeness of the assembly.

 I would recommend that some small editorial changes be made in the "Background' section, as follows:

- change 'type of hoverfly from the Syrphid family'. to  'species of hoverfly in the fly family Syrphidae.'    This provides more formalized language for the sentence.
- Please add a citation or website address for the Darwin Tree of Life project to the last sentence in "Background".

Are sufficient details of methods and materials provided to allow replication by others?

Yes

Is the rationale for creating the dataset(s) clearly described?

Yes

Are the datasets clearly presented in a useable and accessible format?

Yes

Are the protocols appropriate and is the work technically sound?

Yes

Reviewer Expertise:

insect phylogenetics and evolutionary biology

I confirm that I have read this submission and believe that I have an appropriate level of expertise to confirm that it is of an acceptable scientific standard.
